# Synaptic transmission of spike trains with arbitrary interspike intervals

**DOI:** 10.1186/1471-2202-16-S1-P205

**Published:** 2015-12-18

**Authors:** Alex D Bird, Magnus JE Richardson

**Affiliations:** 1 Warwick Systems Biology Centre, University of Warwick, Coventry, UK; 2 School of Life Sciences, University of Warwick, Coventry, UK; 3 Warwick Systems Biology DTC, University of Warwick, Coventry, UK

## 

Short-term synaptic depression, caused by depletion of releasable neurotransmitter vesicles, modulates the strength of neuronal connections in an activity-dependent manner [1,2]. Quantifying the statistics of this form of synaptic transmission requires the development of stochastic models linking probabilistic neurotransmitter release with the spike-train statistics of the presynaptic population [3,4]. A common approach has been to model the presynaptic spike train as either regular or a memory-less Poisson process [5] - few analytical results are available that describe the behaviour of a depressing synapse when the afferent spike train has more complex, temporally correlated statistics.

**Figure 1:**
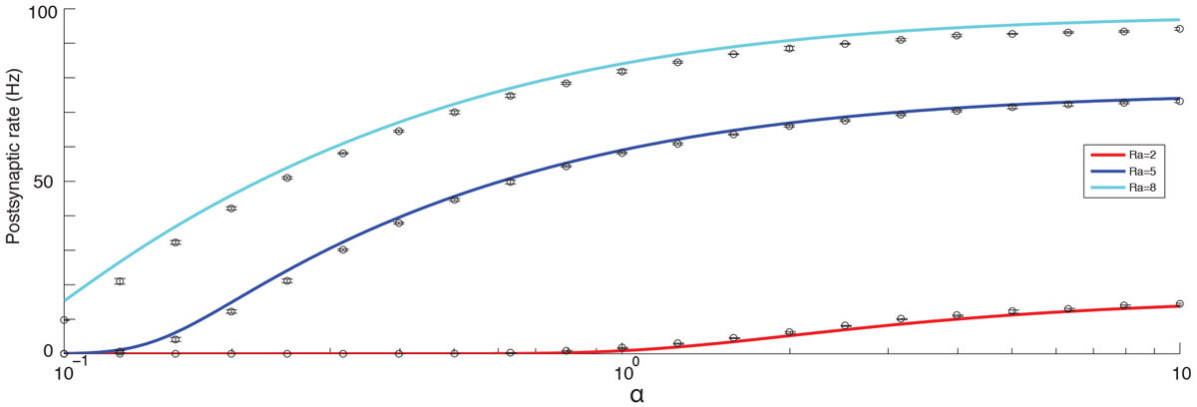
**Predictions of postsynaptic firing rates as a function of the regularity of the renewal process governing presynaptic firing**. Presynaptic interspike intervals are independently identically gamma distributed with shape parameter α (increasing α gives more regular firing) and mean **1/R_α_**. Simulated rates are shown as circles with error bars. Analytic predictions are shown as solid lines based on exact forms for the postsynaptic voltage mean and variance.

Recently, we have derived a series of results that allow for the fraction of occupied release sites and the neurotransmitter release probability to be calculated for a presynaptic spike train with arbitrary interspike interval (ISI) statistics. The results take a particularly compact form when the presynaptic spike times are generated by a renewal process, i.e. when the ISIs are independent. This encompasses a broad range of models that are currently used for circuit and network analyses, including the class of integrate-and-fire models. Our approach also allows for the postsynaptic voltage mean and variance to be calculated, which in turn allows for an approximation of the firing rate of a neuron driven by depressing synapses from non-Poissonian presynaptic neurons (Figure 1).

These results will allow for the incorporation of more complex and physiologically relevant firing patterns into future analytic studies of neuronal circuits and networks.
